# Spike-and-Wave Discharges Are Not Pathological Sleep Spindles, Network-Level Aspects of Age-Dependent Absence Seizure Development in Rats

**DOI:** 10.1523/ENEURO.0253-19.2019

**Published:** 2020-01-02

**Authors:** Gábor Kozák, Tamás Földi, Antal Berényi

**Affiliations:** 1 MTA-SZTE ‘Momentum’ Oscillatory Neuronal Networks Research Group, Department of Physiology, Interdisciplinary Excellence Centre, University of Szeged, Szeged H-6720, Hungary; 2 Neuroscience Institute, New York University, New York City, NY 10016

**Keywords:** absence seizure, maturation, rat, sleep spindle, spike and wave, thalamocortical

## Abstract

Spike-and-wave discharges (SWDs) of absence epilepsy are considered as pathologic alterations of sleep spindles; however, their network-level relationship has never been convincingly revealed. In order to observe the development and generalization of the thalamocortical SWDs and the concomitant alterations of sleep related oscillations, we performed local field potential (LFP) and single unit recordings in rats for three months during their maturation. We found that while SWDs and spindles look similar in young, they become different with maturation and shift to appear in different brain states. Thus, despite being generated by the same network, they are likely two distinct manifestations of the thalamocortical activity. We show that while spindles are already mainly global oscillations, SWDs appear mainly only focally in young. They become capable to generalize later with maturation, when the out-of-focus brain regions develop a decreased inhibitory/excitatory balance. These results suggest that a hyperexcitable focus is not sufficient alone to drive generalized absence seizures. Importantly, we also found the gradual age dependent disappearance of sleep spindles coinciding with the simultaneous gradual emergence of spike and waves, which both could be reversed by the proper dosing of ethosuximide (ETX). Based on these observations we conclude that the absence seizure development might be a multi-step process, which might involve the functional impairment of cortical interneurons and network-level changes that negatively affect sleep quality.

## Significance Statement

Absence seizure development parallels with the deterioration of slow-wave sleep (SWS), which might be reversible with ethosuximide (ETX) in a narrow therapeutic range, but not with early electrical seizure abruption. Thus, by proper dosing, spindles can be pharmaceutically recovered while epileptic activity is suppressed and absence related sleep and learning disturbances may be counterbalanced. We further found that despite the characteristic high voltage rhythmic global ictal EEG patterns the absence seizures are not accompanied with widespread phase locking of neuronal activities.

## Introduction

Absence epilepsy is the most common epilepsy of childhood (Jallon et al., 2001). Spontaneous absence seizures in both humans and genetic rodent models are hypothesized to emerge from a hyperexcitable cortical focus (Holmes et al., 2004; Meeren et al., 2005), which generalizes involving the thalamocortical circuitry, and results in sudden loss of consciousness, behavioral arrest and a typical EEG pattern of spike-and-wave discharges (SWDs; Crunelli and Leresche, 2002; Blumenfeld, 2005). Importantly, the interplay between the cortex and the thalamus also gives rise to major physiologic oscillations such as spindles and δ waves during sleep (Steriade et al., 1993). Although these physiologic and pathologic oscillations can coexist in the same circuitry (Steriade et al., 1986; McCormick and Bal, 1997; Sanchez-Vives and McCormick, 2000; Timofeev and Steriade, 2004), very little is known about how the emergence of seizures influence the healthy sleep-related oscillations (Pinault et al., 2001; Beenhakker and Huguenard, 2009). To our knowledge, no study has investigated yet the evolution of the physiologic thalamocortical oscillations in relation to the emerging seizure activity.

Moreover, while numerous studies have provided important insights into the intraseizure and periseizure dynamics of thalamus and cortex, most of these works limited their scope to the investigation of the putative cortical focus and their thalamic peers (Steriade and Contreras, 1995; Pinault et al., 1998; Polack et al., 2007; McCafferty et al., 2018). Thus, it is largely unexplored how local ictal activity of the putative focus can evolve into fully developed, global absence seizures. Furthermore, the contribution of the out-of-focus cortical areas to the seizure evolution is also understudied. However, these questions are of a particular interest, as a recent study suggest that frequent seizures in absence epilepsy might alter the cortical microcircuitry in the seizure initiation zone (Studer et al., 2019). Thus, as fully developed absence epilepsy is a global phenomenon, it is reasonable to consider and investigate whether the out-of-focus cortical areas undergo changes due to the abundant seizures invading the whole cortex (Kozak, 2019).

To address these issues, we performed a longitudinal study in Long–Evans rats using multisite recording electrodes in multiple cortical areas to determine the evolution of spontaneous seizures and the related cooccurring alterations of sleep architecture. The evolution of absence seizures spans months in Long–Evans rats that capacitates this strain to be an ideal model to investigate how the gradual seizure emergence impacts other oscillations of the thalamocortical circuitry parallel to the progression of the epileptic condition. Additionally, to causally investigate the cortical mechanisms of SWD generalization and to understand how maturation influences seizure susceptibility, we also recorded spiking activity in juvenile (i.e., 2.5–4 months old) and mature (i.e., five to seven months old) rats from out of focus cortical sites during spontaneous and electrically induced seizures employing high-density silicone electrodes.

Here, we show that on the long-term, seizure evolution impacts the occurrence of sleep spindles, which is reversible with pharmacological treatment. Furthermore, we found that in contrast to the general belief, the majority of the cortical neurons are in silence during individual SWDs of the absence seizures; most cortical interneurons are suppressed, and pyramidal cells do not increase their firing rate either. Thus, absence seizures in the cortex might be rather related to a shifted inhibitory/excitatory balance than an increased, hypersynchronous discharge rate of the cortical units.

## Materials and Methods

### Animals

All experiments were performed in accordance with European Union guidelines (2003/65/CE) and the National Institutes of Health Guidelines for the Care and Use of Animals for Experimental Procedures. The experimental protocols were approved by the Ethical Committee for Animal Research at the Albert Szent-Györgyi Medical and Pharmaceutical Center of the University of Szeged (XIV/218/2016). Thirteen male Long–Evans rats were used in the present study.

### Surgery

The animals (male Long–Evans rats) were operated under isoflurane anesthesia. The animals for longitudinal observations (*n* = 5, three months old), animals for pharmacological experiments (*n* = 3, five to seven months) and animals for transcranial electrical stimulation experiments (*n* = 6, five to seven months) were implanted with intracortical recording electrode triplets (interwire spacing, 0.4 mm), which targeted the frontal and parietal cortical areas of both hemispheres and unilaterally the CA1 subfield of the hippocampus, as already reported elsewhere in details (Kozák and Berényi, 2017). For transcranial stimulation experiments, additional stimulation electrodes were implanted over the temporal bone bilaterally (Kozák and Berényi, 2017; Kozák et al., 2018). Unit recordings and intracortical stimulation experiments were performed in rats divided into two age groups: juvenile (2.5–4 months, *n* = 5) and mature (five to seven months, *n* = 4) animals. These age groups are referred to as “juvenile” and “mature” animals, throughout the manuscript. Rats were implanted with tripolar electrodes, which targeted the frontal and parietal cortical areas of the left hemisphere and unilaterally the CA1 subfield of the hippocampus and a silicone probe (Neuronexus, Poly2, 32 channels) with a custom-built microdrive (Vandecasteele et al., 2012) allowing for the vertical adjustment during targeting the prefrontal cortex (AP: +2.7 mm from bregma; ML: +1.5 mm, angled at 10° from the sagittal plane) or motor cortex (AP: –2 mm from bregma, ML: +1.5 mm in the sagittal plane) of the right hemisphere. A custom-built bipolar electrode consisting of two insulated tungsten wires, each peeled at the tip for 200 μm (interwire spacing, 0.5 mm) was implanted in the left neocortex (AP: +2 mm; ML: −2 mm; DV: −1.5 mm from the dura (motor area; Maingret et al., 2016). Miniature stainless-steel screws (serving as reference and ground) were implanted above the cerebellum. A copper mesh (serving as a Faraday cage) was built around the probes and enforced with dental cement.

### Electrophysiological recordings and stimulation

The rats were housed in the recording room individually in plastic cages, the walls were made of clear Plexiglas and food and water were given ad libitum. All recording sessions took place in the same room in 12/12 h light/dark cycles. After recovery from the surgery (minimum 3 d), the maturing rats were connected to the recording system and were observed for three months continuously in their home cage, as reported elsewhere already (Kozák and Berényi, 2017). The neuronal signals were preamplified, amplified (total gain 400×), multiplexed on head and stored after digitalization at 20-kHz sampling rate per channel (KJE-1001, Amplipex).

To investigate the effect of antiepileptic treatment on sleep spindles, animals (*n* = 3) received an intraperitoneal injection of saline (control day) then on the following day an intraperitoneal injection of ethosuximide (ETX; treatment day, 100 mg/kg body weight; Richards et al., 2003). Injections were given at 8 a.m. and animals were monitored for 12 h in daylight. These control-treatment sessions were repeated three times, interleaved with 2-d-long rests between the consecutive sessions to ensure the elimination of ETX between measurements (Bachmann et al., 1988).

To investigate the effect of closed-loop transcranial electrical disruption of seizure activity on sleep patterns, animals implanted with transcranial stimulation and intracortical recording electrodes were connected to a custom programmed unsupervised seizure detection and intervention system (RX-8, Tucker-Davis Technologies), reported elsewhere in details (Kozák and Berényi, 2017). Each detected seizure triggered a charge neutral, triphasic single-pulse (100 ms) stimulation (STG4008; Multi Channel Systems) through the bitemporal stimulation electrodes. The closed-loop (*n* = 3) and open-loop (*n* = 3) stimulated animals received treatment in an alternating fashion: each non-stimulated day (control) was followed by a stimulated day (treatment). The stimulation of each open-loop treated animal was driven by the seizure activity of a randomly chosen closed-loop treated animal.

In intracortical stimulation experiments the optimal stimulation voltage was determined in advance for each animal as the minimum voltage (7–15 V) necessary to reliably induce δ waves during NREM sleep. During stimulation a monophasic single-pulse (0.1 ms) was delivered by an isolated stimulator generator (STG4008; Multi Channel Systems) to the deep layers of the motor cortex (anode: −1.5 mm from the dura, cathode: –1.0 mm from the dura). Animals were recorded in their home cage both in control and stimulation sessions. In stimulation sessions intracortical pulses were delivered every 10 s, with no respect to the behavioral states.

### Data analysis

All analyses were conducted off-line. For aging experiment, a random day recording of each week was included. The raw wide-band signal was low-pass filtered and down-sampled to 1250 Hz to generate local field potential (LFP). SWD episodes were detected from the LFP signals recorded in the somatosensory cortex. Forth order Butterworth filter was applied to the LFP (in ranges of 8–12 and 30–200 Hz). Concurrent threshold-crossings in both range were considered as SWD events (Kozák and Berényi, 2017). Consecutive SWD episodes were merged if they followed each other within 1 s. Events shorter than 1 s were excluded from the analysis. For δ wave and sleep spindle detections, we only used non-θ epochs. Non-θ epochs were detected automatically using the ratio of the power in θ band (5–11 Hz) to the power of nearby bands (1–4 and 12–14 Hz) of hippocampal LFP (Sirota et al., 2008). Threshold was manually adjusted for all animals. To detect global δ waves, the LFPs recorded bilaterally in the somatosensory were filtered (0–6 Hz) and Z-scored. Zero-crossings of the first temporal derivatives were calculated. Consecutive upward-downward-upward zero-crossings within a temporal window of 150 and 500 ms were considered as putative δ events. δ Waves corresponded to epochs where Z-score exceeded 2 at the peak, or exceeded 1 and fell below −1.5 at the end of the event bilaterally (Maingret et al., 2016). δ Waves were only used to identify slow-wave sleep (SWS) sessions and to approximate the depth of SWS (Gervasoni et al., 2004). Segmentation of the recording sessions into SWS, transition from awake/REM to SWS, transition from SWS to awake/REM, and awake/REM was performed as follows. Recordings were segmented into 1-min-long epochs. Those epochs, in which δ wave occurrence rate exceeded 10/min, were considered as SWS. SWS epochs only separated by 1 min or less, were merged. The first minutes of SWS with the preceding awake, non-SWS epochs were labeled as “transition to SWS.” Similarly, we identified the last minutes of SWS sessions and the first minutes of non-SWS, awake as “transition from SWS.” Remaining epochs were labeled as “awake/REM.”

To estimate the movement of the animals’ electromyogram (EMG) was extracted from the intracranial signals by detecting the zero time-lag correlation coefficients (*r*) between 300- and 600-Hz filtered signals recorded at different sites. Pairwise Pearson correlations were calculated between pairs of channels that were minimally 400 μm away from each other. The mean of all pairwise correlations measured in each 0.5-s bin was calculated and recorded as an EMG score (Schomburg et al., 2014). EMG signals were used to separate previously identified awake/REM periods to awake and REM periods (REM periods correspond to low EMG, high θ/δ ratio epochs) and to estimate animals’ activity in awake periods.

Spindle detection was performed as follows. The LFP recorded in the somatosensory cortex was bandpass filtered (10–20 Hz) and Z-scored. Spindles corresponded to epochs where Z-score exceeded 2 for >0.2 s and peaked at >4. Events separated by <0.4 s were merged, and combined events lasting >3 s were discarded (Maingret et al., 2016). The events induced by intracortical stimulation were classified manually. Continuous wavelet spectra and power spectra were calculated in MATLAB using Wavelet Toolbox and Chronux Toolbox (http://chronux.org/), respectively.

The spike sorting was performed using Kilosort with its default settings (Pachitariu et al., 2016) and were manually refined in Phy (Rossant et al., 2016). Putative interneurons and pyramidal cells were discriminated based on their spike widths and autocorrelograms (Csicsvari et al., 1998; Mizuseki et al., 2009). For all individual units, spiking activity in a [–88, +88]-ms window centered around the peak of SWDs were collected and firing rate histograms (0.8-ms time bin) were constructed. The firing rate histograms were Z-scored for each unit individually and smoothed with a Gaussian filter. Raster plots were constructed with all the smoothed Z-scored histograms of individual units.

### Histology

At the end of the experiments, electrolytic lesions were made at the tip of the electrodes to verify their location. Animals were deeply anaesthetized with 1.5 g/kg urethane (intraperitoneal) and transcardially perfused with saline followed by 4% paraformaldehyde and 0.2% picric acid in 0.1 M phosphate buffer saline. After overnight postfixation, 50-μm-thick coronal sections were prepared with vibratome (VT1000S Vibratome, Leica) and stained with 4′,6-diamidino-2-phenylindole dihydrochloride (DAPI; D8417, Sigma-Aldrich).

### Statistics

All statistical analyses were performed in MATLAB (The MathWorks). No statistical methods were used to predetermine sample sizes, but the number of animal and recorded cells were similar to those employed in previous works. All tests were two-tailed. Non-parametric Wilcoxon signed-rank test, Kruskal–Wallis one-way analysis of variance, repeated measures analysis of variance, Kolmogorov–Smirnov test, Rayleigh’s tests were used. To investigate spindle characteristics’ change over time, from each analyzed recording sessions we randomly sampled 500 events of each animal and applied repeated measures ANOVA. To perform similar analysis on seizures, we randomly sampled 200 seizure events in each animal. Box plots represent median and 25th and 75th percentiles, and their whiskers the data range. Outlier values are not displayed on the figures, but they were always included in the statistical analysis. Linear regressions were performed using robust-linear regression and *p* values for linear regressions tests the hypothesis that true correlation exists against a null-correlation. Modulation strength was calculated using mean resultant length of the phases (peaks of spike component of SWDs are at 180°) and units were considered to have phase locking to SWDs when α < 0.05 and κ > 0.1 on Rayleigh’s test of non-uniformity using the circular statistics toolbox provided by P. Berens (Berens, 2009).

## Results

### Development of SWDs with maturation

**Figure 1.**
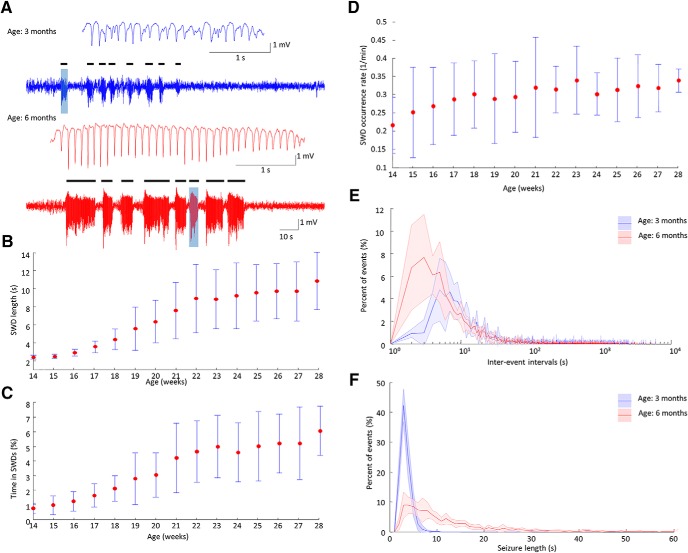
SWD frequency and duration increase with maturation. ***A***, Representative LFP traces of SWD activity at ages of 14 weeks (blue) and 28 weeks (red). Black ticks label individual seizures. Inlets show individual SWD epochs. Group data shows an increasing time spent in SWDs with age, expressed as the percentage of the total observation time [(***B***) *n* = 15 observations per animal in five animals; repeated measures ANOVA, factor: age *F*_(14,56)_ = 13.60; *p* = 0.0210), an increasing average SWD epoch duration; (***C***) *n* = 15 observations per animal in five animals; repeated measures ANOVA, factor: age *F*_(14,56)_ = 18.51; *p* = 0.0126) and increasing SWD rate; (***D***) *n* = 15 observations per animal in five animals; repeated measures ANOVA, factor: age, *F*_(14,56)_ = 2.67; *p* = 0.1773] over the observation period. Error bar represents ±SD, data are sampled for 24 h during each week. Population data shows distribution of interevent intervals (***E***) and the length of SWD epochs (***F***) in the same animals (*n* = 5 animals) at the age of three months (blue) and six months (red), shaded area represents ±SD. Kolmogorov–Smirnov, interevent interval: *p* < 0.001, distribution of seizures, *p* < 0.001; ∗*p* ≤ 0.05, ∗∗*p* ≤ 0.01, ∗∗∗*p* ≤ 0.001.

First, we determined how SWD incidence is influenced by maturation. We found that the total time in SWDs (expressed as the percentage of the 24-h-long recordings spent in SWDs) progressively increased during the observation period and saturated around the age of five months (Fig. 1*A*,*B*), which is in accordance with previous observations of Long–Evans rats reporting stable SWD parameters at the age of six to nine months (Shaw, 2007). SWD epochs were significantly longer in older animals (Fig. 1*C*) and mean length of SWDs showed the same saturation tendency as the total time spent in SWDs. SWD occurrence rate showed an early modest increase that remained steady (Fig. 1*D*). The temporal distribution of the SWDs also changed with age. In six-month-old animals, the median interevent interval was shorter (Fig. 1*E*). This result suggests that in well-developed absence epilepsy bursts of long seizures can be observed, which accounts for most of the time spent in seizures (Fig. 1*F*).

### Relationship between sleep spindles and SWDs

**Figure 2.**
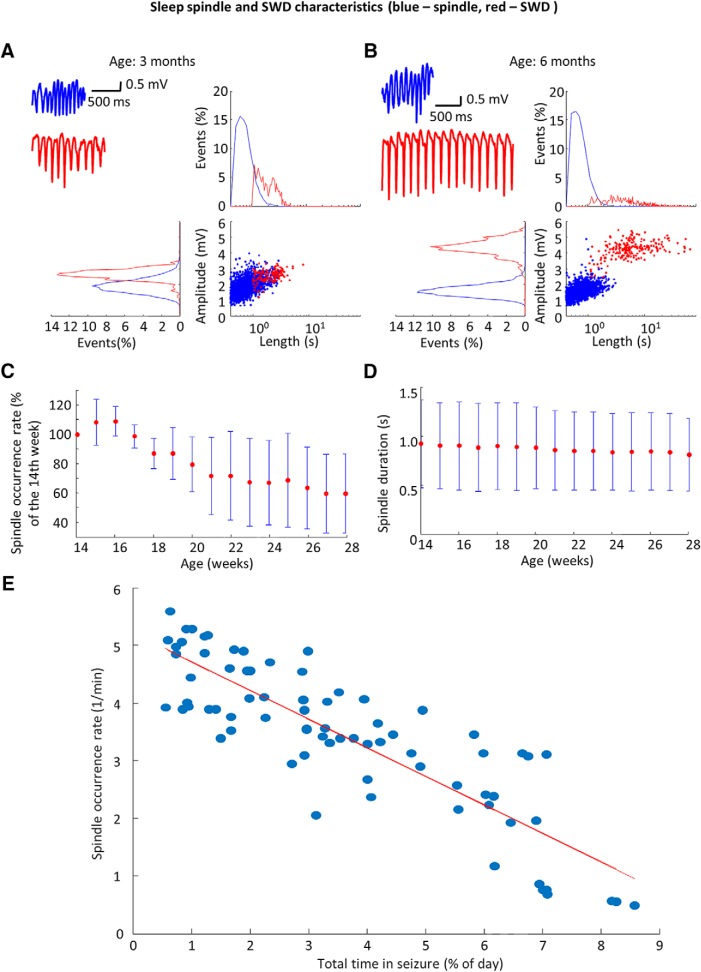
Relationship between sleep spindles and SWDs. ***A***, ***B***, Sleep spindle (blue) and seizure (red) characteristics in a representative animal at three and six months, with example sleep spindles and spike-and-wave episodes (upper left panel). ***C***, ***D***, Population data shows the decrease of sleep spindle duration (*n* = 15 observations per animal in five animals, 500 randomly sampled spindles per each observation were compared; ANOVA factor: age *F*_(14,34930)_ = 14.94; *p* < 0.001) and sleep spindle incidence normalized to the first week of observation (*n* = 15 observations per animal in five animals; ANOVA *F*_(14,56)_ = 7.10; *p* = 0.0562, test was applied before normalization). ***E***, Regression of the total time spent in SWDs and sleep spindle occurrence rate on the population level (*n* = 15 observations per animal in five animals, *R* ^2^ = 0.73; *p* < 0.001; *t* = –13.65), red line corresponds to best linear fit.

As SWDs are often considered as aberrant sleep spindles of the thalamocortical circuitry (McCormick and Bal, 1997; Beenhakker and Huguenard, 2009), we investigated how the progression of SWD-generation impacts physiologic sleep spindles. We found that sleep spindles and SWDs’ duration and amplitude distributions were overlapping at the age of three months, but they became distinct as the animals reached the age of six months (Fig. 2*A*,*B*). The peak spindle frequency did not change with age (data not shown, *n* = 15 observations per animal in five animals, 500 spindles per each observations were compared; ANOVA factor: age *F*_(14,34930)_ = 2.37; *p* = 0.12), however, a gradual fall of spindle occurrence rate (Fig. 2*C*) and duration (Fig. 2*D*) was observed. The increase of total time spent in SWDs, indicative of the progression of the disease, showed strong inverse correlation with the decline of spindle incidence rate that raises the possibility of mutually exclusive mechanisms of spindle and SWD generation. (Fig. 2*E*).

### Effect of antiepileptic treatment on SWDs and sleep spindles

**Figure 3.**
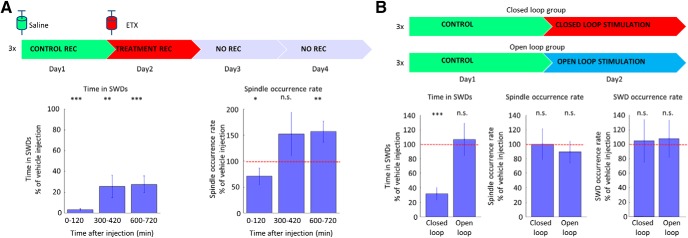
Effect of antiepileptic treatment on SWDs and sleep spindles. ***A***, The effect of ETX treatment on time spent in seizures (left) and on sleep spindle occurrence rate (right), expressed as the percentage of the control days’ values (*n* = 3 control vs treatment session in three animals, Wilcoxon signed-rank test, control vs treatment). ***B***, The effect of closed loop and open loop transcranial electrical stimulation on time spent in seizures, on sleep spindle occurrence rate, and on the seizure occurrence rate (*n* = 3 control vs treatment session in three animals, Wilcoxon signed-rank test, control vs treatment; data from Kozák and Berényi, 2017) expressed an the percentage of the control days’ values. Error bars represent SEM; ∗*p* ≤ 0.05, ∗∗*p* ≤ 0.01, ∗∗∗*p* ≤ 0.001.

Next, we investigated the effect of ETX, the drug of first choice in absence epilepsy, on sleep spindle occurrence (Fig. 3*A*). A single high dose injection of ETX (100 mg/kg body weight; Richards et al., 2003) resulted in a prompt SWD suppression (0–120 min after injection, 3.35 ± 3.76% of the vehicle injection), which was accompanied with a reduction in sleep spindle occurrence (0–120 min after injection, 70.80 ± 45.46% of the vehicle injection), too. Later, as the drug’s plasma level decreased, ETX still had the potential to suppress SWD activity (600–720 min after injection, 27.62 ± 24.27% of the vehicle injection), but simultaneously sleep spindle occurrence was higher than the time-matched baseline activity of vehicle injection (600–720 min after injection, 156.72 ± 60.18% of the vehicle injection). To further investigate, whether this increased spindle occurrence rate was only the mechanistic consequence of the increased SWD-free time of the animals, we disrupted spike-and-wave activity with on-demand transcranial electrical stimulation in three animals. We found, that although transcranial electrical stimulation was effective in quickly terminating the SWDs (*n* = 3 animals, as the total time spent in SWDs decreased), it did not decrease their incidence, and did not influence positively sleep quality by leaving sleep spindle occurrence rate unchanged (Fig. 3*B*).

### Brain state dependence of spontaneous SWDs

**Figure 4.**
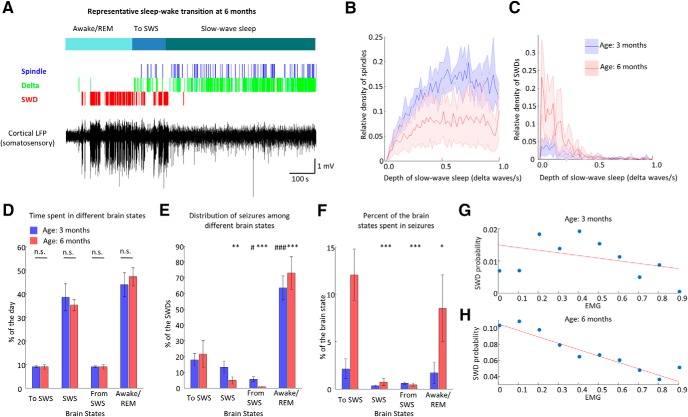
Brain state dependence of spontaneous SWDs. ***A***, Representative wake-sleep transition of a mature rat. Black trace is the LFP of the somatosensory cortex, colored ticks highlight individual SWDs (red), δ waves (blue), and sleep spindles (green), classified brain states are showed on top in shades of blue. ***B***, ***C***, Relative occurrence of sleep spindles and SWDs as the function of δ wave density at three months (blue) and six months (red; Kolmogorov–Smirnov, sleep spindles: *p* < 0.001, seizures: *p* < 0.05). ***D***, Population data shows the time spent in different brain states at three months (blue) and six months (red) age in the same animals (*n* = 5 animals, two-way ANOVA, interaction between age and brain state, *F*_(3,32)_ = 1.9; *p* = 0.1492). ***E***, Population data shows the distribution of SWD epochs among different brain states in the same animals (*n* = 5 animals, two-way ANOVA, interaction between age and brain state, *F*_(3,32)_ = 4.78, *p* = 0.0073; *post hoc* Tukey–Kramer; vs three months To SWS: #*p* ≤ 0.05; ###*p* ≤ 0.001; vs six months to SWS: ∗∗*p* ≤ 0.01, ∗∗∗*p* ≤ 0.001). ***F***, Population data shows the percentage of time spent in SWDs in different brain states (*n* = 5 animals, two-way ANOVA, interaction between age and brain state, *F*_(3,32)_ = 21.78, *p* < 0.001; *post hoc* Tukey–Kramer, vs six months to SWS: ∗*p* ≤ 0.05, ∗∗∗*p* ≤ 0.001). ***G***, ***H***, Probability of SWD occurrence in awake animals as the function of preseizure normalized EMG signal at three and six months of age in the same animals. Red lines, best linear fit (*n* = 5 animals, age: three months: *R* ^2^ = 0.1554; *p* = 0.2991; *t* = –1.1104; age: six months: *R* ^2^ = 0.8942; *p* < 0.001; *t* = –7.8231).

Next, to further clarify the relationship of spontaneously emerging SWDs and physiologic thalamocortical oscillations (Beenhakker and Huguenard, 2009), we investigated how these patterns are generated around sleep. The incidence of spontaneous sleep spindles and SWDs with respect to the depth of the SWS (approximated by the density of δ waves, where higher density of δ waves corresponded to deeper SWS; Gervasoni et al., 2004; Lacroix et al., 2018) was different. Unlike sleep spindles, SWDs emerged at the transitions between wakefulness and sleep and their occurrence rate progressively fell with deepening of the SWS (Fig. 4*A–C*). Next, we analyzed the brain state dependence of SWD occurrence. Segmenting the recordings into SWS, transition to SWS, transition from SWS (2-min-long epochs with 1-min SWS and 1-min awake/REM period), and the rest as awake/REM sleep (Materials and Methods; Fig. 4*D*), we found that the SWD occurrence showed a substantial asymmetry regarding SWS (Fig. 4*E*). SWDs were likely to occur during transitions to SWS, but only few SWD epochs were observed during SWS and during transitions from SWS.

In absolute terms, the majority of the SWDs occurred in awake/REM state, but taking into account the time spent in different brain states, the transition to SWS found to be similarly susceptible to SWD generation (Fig. 4*F*). Next, based on derived EMG signals (see Materials and Methods), we removed from the previously identified Awake/REM periods the REM sleep to investigate SWD susceptibility only in awake periods (REM corresponds to low EMG values with high θ/δ ratio; Materials and Methods). and we found that SWDs were more likely to occur when the animals were not active (Fig. 4*G*,*H*), in accordance with previous findings (Marescaux et al., 1992; Steriade et al., 1993; Shaw, 2004; Smyk et al., 2019). Altogether, animals were susceptible to SWDs mainly in inactive resting wakefulness and when they were sleepy.

### Topographic differences and characteristics of SWDs

Originally, absence epilepsy was considered as an instantaneously generalizing, global seizure type, but to date there is an increasing body of evidence suggesting that SWDs emerge locally from a cortical focus located in the somatosensory cortex (Meeren et al., 2002; Polack et al., 2007; Crunelli et al., 2011). Most currently available studies have investigated only the putative initiation zone of the ictal activity (Polack et al., 2007; Williams et al., 2016; Jarre et al., 2017), but have not clarified how other cortical areas are invaded or being surpassed by the SWDs.

**Figure 5.**
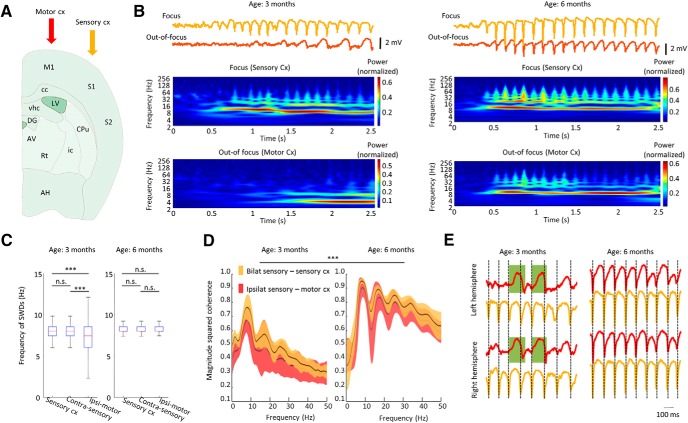
Topographic differences and characteristics of SWDs. ***A***, Typical arrangement of unilateral recording electrodes in the putative “initiation zone” (orange) and in the adjacent motor cortex (red). M1, primary motor cx; S1, primary somatosensory cx; S2, secondary somatosensory cx; CPu, caudate putamen; DG, dentate gyrus; AV, anteroventral thalamus; Rt, reticular thalamic nucleus; vhc, ventral hippocampal commissure; LV, lateral ventricle; cc, corpus callosum; ic, internal capsule; AH, anterior hypothalamic area. ***B***, Representative LFP traces and corresponding wavelet spectrum of typical intraseizure activity of these cortical areas. ***C***, Population data shows mean frequency of spike and wave episodes in different cortical recording sites (ipsilateral and contralateral somatosensory cortices and ipsilateral motor cortex) at three and six months (*n* = 2 observations per animal in five animals, 200 randomly sampled SWDs epochs per each observation were compared). ***D***, Population data shows increasing coherence of intraseizure LFP signals from three months to six months of age between different cortical recording sites in the same animals [bilateral somatosensory cortices (orange), ipsilateral somatosensory and motor cortices (red), *n* = 15 observations per animal in five animals, two-way repeated measures ANOVA on peak coherence of intra-SWD LFP, factor: age *F*_(14,112)_ = 44.918; *p* < 0.001; factor: region (focus, off-focus), *F*_(14,112)_ = 0.57076; *p* = 0.4716]. ***E***, Representative LFP traces (orange, somatosensory cortex; red, motor cortex) show intraseizure dynamics at three and six months in the same animal. Dashed lines refer to the peak of SWDs, δ wave-like activity of motor cortices are highlighted; ∗*p* ≤ 0.05, ∗∗*p* ≤ 0.01, ∗∗∗*p* ≤ 0.001.

Therefore, we systematically investigated SWDs emerging in the somatosensory cortex, on the neuronal activity of out-of-focus cortical areas. First, we compared the LFPs of the motor and the somatosensory cortices of the maturing rats during SWD epochs (Fig. 5*A*). The ictal LFP spectra of the different areas revealed that at the age of three months the somatosensory cortex already expressed SWD oscillations (8.20 ± 2.10 Hz; Fig. 5*B*), characteristic of SWDs. On the other hand, the motor cortex displayed a slower frequency oscillation (7.36 ± 2.29 Hz), that gradually increased its frequency with maturation to reach the main SWD frequency (8.23 ± 0.78 Hz; Fig. 5*C*). Importantly, the bilateral somatosensory cortices showed higher coherence at the age of three months than the unilateral adjacent structures, but at the age of six months seizures became widely coherent with LFP activity locked to the SWD frequency (Fig. 5*D*). An interesting observation is that at the age of three months the characteristic frequency of the off-focus motor cortex often fell in the range of SWS during seizures in the somatosensory cortex and even δ waves were present (Fig. 5*E*).

### Firing dynamics of single units around SWDs

**Figure 6.**
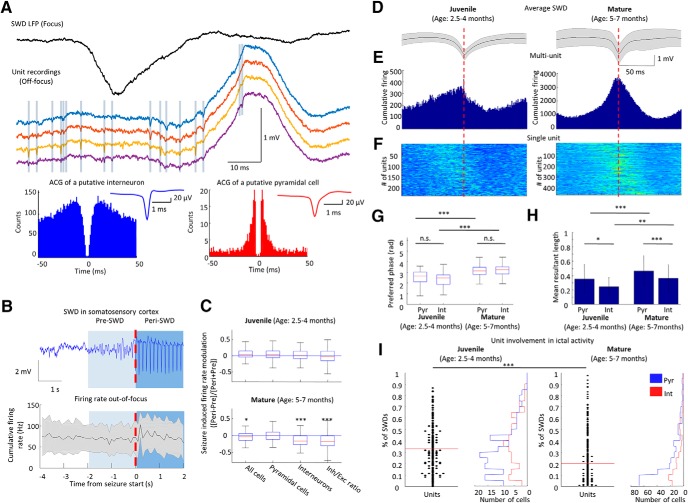
Firing dynamics of single units around SWDs. ***A***, LFP trace of somatosensory cortex and total firing rate of motor cortex around seizure initiation. ***B***, Boxplot of firing rate ratios of units (all cells, pyramidal cells, interneurons and inhibitory/excitatory ratio) before and following the SWD onset ([Peri-Pre]/[Peri+Pre]; Wilcoxon signed-rank test, pre-SWD vs SWD, juvenile: *n* = 118 pyramidal cells and *n* = 77 interneurons, respectively, in 126 events of three animals; total *p* = 0.3136; *Z* = 1.01, excitatory *p* = 0.4542; *Z* = 0.75, inhibitory *p* = 0.6986; *Z* = 0.39, inhibitory/excitatory *p* = 0.82485; *Z* = –0.22; mature: *n* = 318 pyramidal cells and 113 interneurons, respectively, in 215 events of four animals, total *p* < 0.05; *Z* = –2.26, excitatory *p* = 0.9832; *Z* = 0.02, inhibitory *p* < 0.001; *Z* = –3.98, inhibitory/excitatory *p* < 0.001; *Z* = –5.93). ***C***, Average SWDs at different ages. ***D***, All unit and multiunit firing around SWDs. ***E***, Unit activity around SWDs. ***F***, Phase distribution of all spikes around SWDs. Peak of spike component corresponds to 180°. ***G***, ***H***, Phase preference and coupling strength of unit firing around SWDs for modulated interneurons and pyramidal cells [Kruskal–Wallis one-way analysis of variance, *post hoc* Wilcoxon signed-rank test, juvenile: *n* = 115/225 (82/143 pyramidal cells, 33/82 interneurons) in three animals, mature: *n* = 351/408 (252/293 pyramidal cells, 99/115 interneurons) in four animals, phase preference: *F*_(3,462)_ = 50.64 *p* < 0.001; mean resultant length: *F*_(3,462)_ = 72.57 *p* < 0.001]. ***I***, Unit involvement in ictal activity at each SWDs are plotted as percentage of SWDs in which they fire for each unit (left) and as the distribution of cell type-specific contribution [right, blue: pyramidal cell, red: interneuron; Wilcoxon signed-rank test, juvenile (*n* = 225 units) vs mature (*n* = 408 units) *Z* = 8.30; *p* < 0.001]. Red line represents mean; ∗*p* ≤ 0.05, ∗∗*p* ≤ 0.01, ∗∗∗*p* ≤ 0.001.

Recent technical improvements made it possible to observe cortical network dynamics around seizures and an increasing body of evidence suggest that hypersynchronous SWDs are not necessarily accompanied by cellular level hyperactivity (Carney and Jackson, 2014; McCafferty et al., 2018; Meyer et al., 2018) To understand how off-focus cortical neurons contribute to generalizing SWD-patterns, we investigated how neuronal spiking of motor and prefrontal cortices were related to the spontaneously emerging SWDs (Fig. 6*A*). We compared the firing patterns during seizure emergence by looking at the firing rate of neurons (all neurons, putative excitatory pyramidal cells, putative inhibitory interneurons and inhibitory/excitatory ratio) in a 2-s windows prior and following the seizure onset (Fig. 6*B*). During the preictal periods, the firing rate relationship of the putative excitatory cells (i.e., pyramidal cells) and the putative inhibitory cells (i.e., interneurons) did not change with maturation (firing rates of 118 vs 318 pyramidal cells, *Z* = 1.05, *p* = 0.30; 77 vs 113 interneurons’ firing rate, *Z* = 1.69, *p* = 0.09, from juvenile and mature animals, respectively, Wilcoxon signed-rank test), suggesting no gross change in the overall inhibitory/excitatory balance under non-ictal conditions. During the SWDs though, the firing rate of pyramidal cells did not change, while interneurons in mature animals with well-developed SWDs were less active compared to the pre-SWD baseline activity (in juveniles no difference was observed), causing the inhibitory/excitatory balance to shift toward excitation (Fig. 5*C*).

We next asked whether cells were entrained to seizure activity on a cycle-by-cycle basis or the LFP pattern of SWDs was more likely an emergent network property with loosely coupled unit activity, as it was reported in the thalamus (Buzsaki, 1991; Steriade and Contreras, 1995; McCafferty et al., 2018). We found that 53.3% (42.7% of interneurons and 59.4% of pyramidal cells) of units in juvenile and 88% (89.6% of interneurons and 87.3% of pyramidal cells) of mature animals’ units showed phase locking to ongoing seizure activity (Fig. 6*D–H*). Interestingly, the preferred phase showed no cell-specific difference, but the pyramidal cells were significantly stronger coupled to SWDs (Fig. 6*G*,*H*). Furthermore, most cells were only firing in ∼25% of the total SWD cycles (Fig. 6*I*). It is possible, that since thalamocortical cells are not entrained in every cycle of the ictal activity (McCafferty et al., 2018), they do not provide thalamic feedback to their cortical counterparts on a cycle-to-cycle basis, and thus, most of the cortical cells are not constrained at each discharge even when an SWD is generalized.

### State-dependent seizure susceptibility

**Figure 7.**
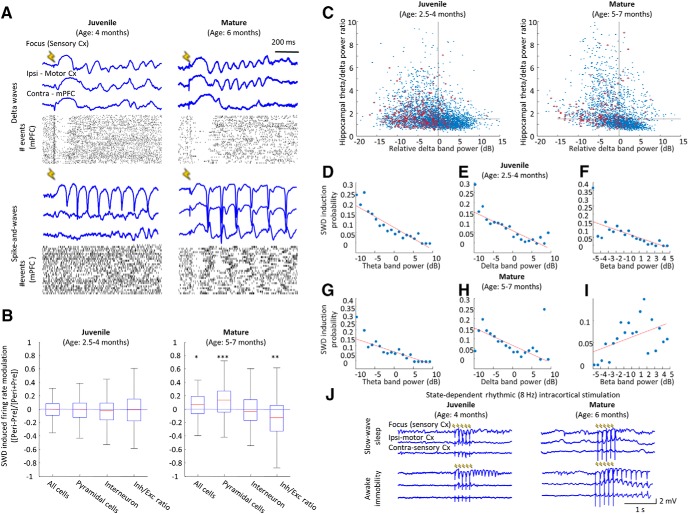
State-dependent seizure susceptibility. ***A***, Representative LFP traces (single event) of different cortical areas and raster plot of cortical units (mPFC) during stimulation trials, which successfully induced δ waves or spike and waves in a juvenile and a mature animal. ***B***, Boxplot of firing rate ratios of units (all cells, pyramidal cells, interneurons and inhibitory/excitatory ratio) around successful seizure induction ([Peri-Pre]/[Peri+Pre]; Wilcoxon signed-rank test, preseizure vs seizure, juvenile: *n* = 107 pyramidal cell and *n* = 60 interneurons, respectively, in three animals; total *p* = 0.8319; *Z* = –0.21, excitatory *p* = 0.564; *Z* = –0.58, inhibitory *p* = 0.5366; *Z* = –0.62, inhibitory/excitatory *p* = 0.74743; *Z* = –0.32; mature: *n* = 317 pyramidal cells and *n* = 117 interneurons in four animals, total *p* < 0.05; *Z* = 2.28, excitatory *p* < 0.001; *Z* = 3.48, inhibitory *p* = 0.4969; *Z* = –0.68, inhibitory/excitatory *p* < 0.01; *Z* = –3.01). ***C***, Brain state dependence of single-pulse stimulations. Each blue dot represents a single trial as the function of cortical δ band power and hippocampal θ/δ ratio. Dashed lines divide trials into four panels. Lower right panel corresponds to SWS. Red circles denote successful seizure inductions (juvenile: *n* = 10 sessions in five animals, mature: *n* = 7 sessions in four animals). ***D***, ***G***, Population data shows probability of SWD induction as the function of prestimulus hippocampal θ band activity; (***E***, ***H***) prestimulus cortical δ band activity and (***F***, ***I***) prestimulus cortical β band activity. Red lines: best linear fit (juvenile: *n* = 10 sessions in five animals, mature: *n* = 7 sessions in four animals; juvenile, θ: *R* ^2^ = 0.8425; *p* < 0.001; *t* = –9.6547; δ: *R* ^2^ = 0.7205; *p* < 0.001; *t* = –8.8036; β: *R* ^2^ = 0.4724; *p* < 0.001; *t* = –6.3559; mature, θ: *R* ^2^ = 0.6645; *p* < 0.001; *t* = –6.4961; δ: *R* ^2^ = 0.1416; *p* < 0.001; *t* = –6.3944; β: *R* ^2^ = 0.2363; *p* < 0.05; *t* = 2.2121). ***J***, Effect of 8-Hz intracortical stimulation in juvenile and mature rats during NREM sleep and in awake state; ∗*p* ≤ 0.05, ∗∗*p* ≤ 0.01, ∗∗∗*p* ≤ 0.001.

Furthermore, to investigate SWD susceptibility, we tested how single-pulse intracortical electrical stimulation can influence brain activity. It is known that short single-pulse stimulation during NREM sleep can induce global δ waves, which are identical to the spontaneously emerging ones (Vyazovskiy et al., 2009; Maingret et al., 2016). Mechanistically, such electrical stimulation causes the simultaneous depolarization of some pyramidal cells, which in turn can entrain distant cortical regions into sleep oscillation via corticocortical connections (Vyazovskiy et al., 2009). Given that single-pulse stimulation has the potential to elicit global sleep patterns, we asked whether it is also sufficient to switch the brain into seizure activity. After determining the minimal intensity required to induce δ waves during SWS (Materials and Methods), we applied the same intensity stimulation during each brain state. Indeed, we were able to induce δ waves successfully as reflected by their characteristic LFP and unit-firing patterns (Fig. 7*A*). Interestingly, both juvenile and mature animals’ somatosensory cortices could respond with spike-and-wave activity to the stimulation, although juvenile animals’ ictal-like activity often remained local, but mature animals developed generalized seizures.

Next, we compared spiking activity around electrical stimuli to see how promptly the cortex can react to stimulation. to do that, we compared the firing rate of units (total, excitatory, inhibitory and inhibitory/excitatory) in 1-s windows prior and following stimuli. In juvenile animals, no change was observed in the investigated parameters, but in mature ones, total and excitatory firing rate increased after stimulation, therefore the inhibitory/excitatory balance shifted similarly to the spontaneously emerging seizures (Fig. 7*B*). Next, we determined the susceptibility of cortex for seizures in various brain states (Fig. 7*C*). In accordance with the spontaneously emerging seizures, stimulation during SWS were not able to induce seizure activity neither in juvenile nor in mature animals, but other brain states were susceptible for seizure induction. to determine what makes the cortex susceptible for seizure induction, we tested the correlation of seizure induction efficacy and the prestimulation power of characteristic oscillations (hippocampal θ, cortical β and δ). We found that both hippocampal θ power and cortical δ power negatively correlated with seizure induction probability (Fig. 7*D*,*E*,*G*,*H*). The latter relationship is in line with the rare occurrence of spontaneous seizures and low yield of electrical triggering during NREM sleep. Interestingly, we found a different seizure susceptibility regarding cortical β band. In juveniles, β power negatively correlated with seizure induction probability, but in contrast, it showed positive correlation in mature animals (Fig. 7*F*,*I*).

Finally, we applied a rhythmic 8 Hz cortical stimulation to mimic the activity of a seizure focus in sleeping and in awake animals. We found that regardless the age, even rhythmic stimulation fails to induce sustainable seizures and immediately after stimulation withdrawal, cortex keeps on generating sleep-related patterns. On the contrary, in awake animals these stimulations easily induced seizures in both juvenile and mature animals (Fig. 7*J*). This finding further supports the importance of brain state in seizure susceptibility.

## Discussion

To our knowledge, this is the first study, which follows the evolution of thalamocortical rhythms in the same animals over the time course of three months in multiple cortical locations. We showed that maturation gradually shifts local ictal activity of the somatosensory cortex into global SWDs of the thalamocortical circuitry, which parallels the progressive disappearance of sleep spindles. Importantly, the sleep related changes seem to be reversible by pharmacological therapy. Furthermore, we showed that even a single cortical depolarization burst is sufficient to kick a susceptible brain into seizure activity.

### Animal models of absence epilepsy

Before discussing the implications of our findings for absence epilepsy, it is important to consider some issues related to the rodent models of absence epilepsy. Beside the inbred genetic models of absence epilepsy (WAG/Rij, GAERS; Coenen and Van Luijtelaar, 1987; Marescaux et al., 1992), it is known that Long–Evans (and other outbred strains, such as Wistar and Sprague Dawley) rats can express SWDs with similarity to absence seizures on the cellular (Polack and Charpier, 2006), behavioral (Semba et al., 1980) and pharmacological (Shaw, 2004) levels. Interestingly, the WAG/Rij, GAERS and Long–Evans strains show different age dependence of seizure development (Coenen and Van Luijtelaar, 1987; Jarre et al., 2017). The relative late appearance of absence seizures in Long–Evans rats made the continuous long-term recordings possible, as the growth of their skull is less during the observation period, thus they did not grow out the implants. Importantly, none of the rodent models of absence epilepsy can resemble the frequent spontaneous disappearance of seizures observed in humans (Crunelli and Leresche, 2002), thus there is an ongoing debate about how much the rodent SWDs can be used as a model to human absence epilepsy (Taylor et al., 2017). Most studies confirm the face validity, pharmacological predictive value and construct validity of rats in absence research (Depaulis and Charpier, 2017), thus, we consider the gradual development of the disease in the Long–Evans strain as an ideal temporal window to observe the sequence of network level changes at the cellular and network levels that may help to understand how seizures develop in human, too.

### Absence epilepsy and sleep spindles

It is known, that absence epilepsy is very often accompanied with sleep disturbances in human (Myatchin and Lagae, 2007) and the progressive disappearance of sleep spindles might account for an impairment of learning abilities (Radek et al., 1994; Nolan et al., 2004), but the exact mechanisms are still elusive. We found that sleep architecture has changed during maturation. The NREM sleep became less spindle rich, as the spindle occurrence markedly decreased, but the peak frequency of the individual spindles did not change over the observation period. Altogether these findings raise the possibility of an impaired spindle initiation. Here, we showed that the parallel disappearance of sleep spindles and the emergence of spike and waves might be two joint consequences of the same underlying mechanism even if they affect different phases of sleep, as the treatment of absence epilepsy with ETX can increase the occurrence rate of sleep spindles in a dose-dependent manner. Similar results were found in human (Kellaway et al., 1990), although no systematic clinical study on antiepileptic drugs confirmed this effect of ETX treatment yet. The initial suppression of spindles is understandable as high dose of ETX might critically decrease the availability of T-type Ca^2+^ channels necessary for spindle initiation, but smaller plasma concentrations of ETX resulted in reduced seizure activity and high spindle occurrence. ETX exerts its pharmacological effect mainly via blocking T-type Ca^2+^ channels, although it has other targets as well and its seizure suppression effect is not confined to the thalamus (Manning et al., 2004). Thus, it has to be further investigated whether the frequent seizing is impairing the thalamocortical circuitry (Kozak, 2019) or a maturation-related cellular alteration may be in favor of seizure spread and causing spindle initiation difficulties in an already epileptic brain. Nevertheless, the restoration of sleep spindles by the antiepileptic drug of ETX suggests a network effect of channel deficiencies which causes spindle initiation difficulties in the epileptic brain, especially since the on-demand seizure control did not have similar positive effect on sleep spindles.

### Cortical single-cell activity during SWDs

An interesting aspect of the results is that most cortical units, similarly to their thalamic counterparts (Buzsaki, 1991; McCafferty et al., 2018), are not firing during most of the spike and waves and altogether a distributed firing pattern of always-changing set of spike-and-wave entrained ensembles was observed during ictal activity with a decreased inhibitory/excitatory balance. Furthermore, it is possible that the symptoms of absence epilepsy, subjects being non-responsive for environmental stimuli during the seizures, are connected not only to the generalized synchronous activity of the minority of the units, but to the silence of the majority at the same time. Some studies showed intact cortical processing and partial consciousness during absence seizures in animals (Drinkenburg et al., 2003) and in human as well (Chipaux et al., 2013; Guo et al., 2016). The underlying reason for this might be that in each cycle, most cortical and thalamic units are not entrained to seizure activity, therefore the unaffected loops can still process incoming information from the external world and can explain why epileptic activity can be interrupted by unexpected sensory stimuli.

The overall decrease of firing of the inhibitory cells suggests that the silence of the units is not due to the powerful inhibition on the pyramidal cells but rather due to their weaker thalamocortical drive. On the other hand, our results show a higher inhibitory/excitatory balance in juvenile animals during ictal activity compared to mature ones, what raises the possibility of an active cortical inhibitory veto of seizure spread that disappears with age. Indeed, there is evidence that epileptic animals have an impairment of intracortical GABAergic inhibition (Luhmann et al., 1995; Crunelli et al., 2011). Importantly, our observations are in line with the results of Meyer et al. (2018), who showed using calcium imaging in the visual cortex of stargazer mice, that most inhibitory cells decrease their activity during absence seizures and most cells are not entrained to all of the SWD cycles. Additionally, a recent study investigating the activity of cortical pyramidal cells found that even in the seizure initiation zone pyramidal cells do not increase their firing rate during seizures (McCafferty et al., 2018).

An alternative mechanistic explanation of the decreased inhibitory activity is that while the bursts of the otherwise sparsely firing pyramidal cells during the spikes can counterbalance their decreased activity during the waves resulting in no gross change in their average firing rate, the already fast-firing interneurons cannot further increase their activity, and thus cannot compensate for their relative suppression during the wave phases. This speculation suggests that the decreased interneuron activity is a mere consequence of the altered rhythmicity, and thus, it may help to maintain the SW patterns but may not encounter for the increased susceptibility for seizure invasion of the given region. Further investigations are needed to clarify this question of mutual causality.

### Generalization of SWDs

It is noteworthy, that while spindles are mainly global patterns already in the juvenile animals, the majority of both the spontaneous or induced ictal activity are local without generalization. This underscores that the hyperexcitable focus per se is not sufficient to drive generalized seizures, although the thalamocortical circuitry is already capable to generate generalized patterns. However, during maturation some yet undetermined changes happen which turn the whole brain to be susceptible for epileptic seizures. The hyperexcitability of the cortical focus develops earlier than the propensity of the adjacent areas to participate in the ictal activity, which suggests that seizure development is a multiple-step process.

A study showing that the unilateral inactivation of thalamus can disrupt epileptic seizures ipsilaterally presented similar cortical traces during contralateral seizure activity than we report here in juvenile animals (Buzsaki et al., 1988). As the cortical spread of ictal activity is hypothesized to happen through corticocortical connections, this highlights the importance of thalamocortical feedback on cortical units which frames them to fire synchronously to the seizure drive. Furthermore, the observed δ wave like activity of the motor cortex in response to the ongoing seizure activity of the somatosensory cortex in juvenile animals taken together with the very strong state dependence of seizure susceptibility raises the possibility that in juvenile animals the different cortical areas might be concomitantly in different microstates. Sleep studies reported that cortical areas can selectively go “off-line,” a phenomenon known as local sleep (Vyazovskiy et al., 2011). Therefore, it is possible that the differential upstream determinants of cortical states locally can result in different response to incoming seizure drive, as one can develop a spike-and-wave and the other skips a cycle of the seizure with entering a δ wave. The existence of microstates reflects the independence of local thalamocortical loops to some extent and it may be possible that maturation increases the intrathalamic synchrony resulting in a more uniform response to cortical stimulation and generalization. A previous study already showed that ion channel loss in the thalamic reticular nucleus (TRN) cause the hypersynchrony of TRN neurons, leading to absence seizures (Makinson et al., 2017). Although it is important to mention that in such a multiple-step process a slight imbalanced shift to higher synchrony together with the already hyperexcitable cortex might probably be sufficient to initiate and maintain generalized seizures.

### State dependence of seizure susceptibility

We showed here that spontaneous seizure occurrence correlates with changes in wakefulness. While the seizure probability is very high around transitions between wakefulness and light sleep, seizures are replaced by slow waves as sleep deepens and even rhythmic cortical activation fails to induce seizures in this state. It is known that arousal related brain states are controlled locally via the locus coeruleus noradrenergic system (Constantinople and Bruno, 2011). Furthermore, during transition to SWS the synchrony among TRN neurons is increased which is physiologically responsible for gating the sensory information to the cortex (Halassa et al., 2014). Alternatively, this also can be in favor of spreading highly synchronous activity of epileptic seizures (Makinson et al., 2017). It is possible that in maturing animals during wake-sleep transitions the thalamic synchrony is imbalanced in a way that the continuously decreasing arousal together with increasing TRN synchrony provides a window where the cortical excitability is still high enough to initiate a putative seizure and the TRN is already sufficiently synchronous to provide a sufficiently broad feedback to the cortex via the thalamocortical cells to frame seizure activity for the next cycle. We found that seizure susceptibility oppositely correlates with cortical β oscillations in juvenile and in mature animals. The opposite correlation raises the possibility that these β oscillations have different origin depending on the age of the animals. In juvenile animals it might represent physiologic β oscillation, which is associated with attention and wakefulness, therefore negative correlation with seizure susceptibility is understandable. On the contrary, in mature animals, pathologic β oscillation (Grandi et al., 2018) may emerge, which can contribute to seizure susceptibility. A recent study using optogenetic thalamic stimulation to induce seizures (Sorokin et al., 2016) described similar relationship between β power and seizure induction probability as we found in mature rats. As they hypothesized, this cortical β oscillation might synchronize and frame TRN firing to be pro-epileptic and making generalization of seizures possible.

We propose here that the development of absence epilepsy in polygenic models requires multiple steps, which very likely includes the development of a hyperexcitable cortical initiation zone and the occasional imbalance between thalamocortical synchrony and cortical excitability, which might be present in the form of pathologic β oscillation. Altogether these multiple minor alterations might turn the thalamocortical dynamics to favor seizure spread in polygenic animal models and human subjects.

## Synthesis

Reviewing Editor: Jorge J. Palop, Gladstone Institutes and UCSF Decisions are customarily a result of the Reviewing Editor and the peer reviewers coming together and discussing their recommendations until a consensus is reached. When revisions are invited, a fact-based synthesis statement explaining their decision and outlining what is needed to prepare a revision will be listed below. The following reviewer(s) agreed to reveal their identity: Jordan Sorokin. Note: If this manuscript was transferred from JNeurosci and a decision was made to accept the manuscript without peer review, a brief statement to this effect will instead be what is listed below.

Synthesis:

The authors investigate the progression of absence seizure dynamics in the Long Evans rat model of absence epilepsy, and characterize the relationship between spike wave discharges (SWDs), spindle waves, and brain-state. Using local field potential from within-focus (somatosensory), and out-of-focus (motor) cortical regions, they find that SWDs show a progressive recruitment of non-focal cortical regions during maturation. They also show that, while early in development SWDs and spindles display similar amplitudes and dynamics, by maturation the two thalamocortical events separate both in their amplitudes and in their dependence on brain state. Seizures become unlikely during slow-wave sleep (SWS), where spindles are prevalent. Moreover, the rate of occurrence of seizures shows an inverse relationship with spindles. The authors go on to characterize the propensity of seizure induction using electrical stimulation, and provide some characteristics of inhibitory and excitatory phase locking with spike-wave discharges.

The reviewers agree that the manuscript provides a detailed longitudinal study of the development of spike wave discharges (SWDs) and spindles in Long Evans rats, which has not been well characterized before. The paper offers interesting novel findings, such as the separation of spindle and SWDs dynamics during maturation, and the gradual recruitment of extra-focal cortical regions. It also identified presumed underlying changes in circuit excitability been explored with LFP and single unit recordings. Thus, there are a number of interesting observations to be reported. However, there were multiple concerns regarding the clarity of the description, interpretation of some of the results, and the conclusions drawn from such small sample sizes.

Previous comments from JN that were not addressed:

Unfortunately, many critical issues raised by previous reviewers at JN have been not been addressed. The authors' rebuttal focused on challenging the reviewers' comments instead of understanding the underlying issues to improve the clarity of the manuscript. As a result, many of the comments have not been addressed, including:

1) Increase the organization and presentation of the manuscript. Add subheading in the result section to match the figures, introduce the question/gap of knowledge to be addressed in each subheading, add conclusions, etc. The result section needs to have a basic structure of a manuscript that is currently missing.

2) Confusion about terminology. SWDs and seizures are still used interchangeable, which creates confusion. Please use “SWDs” since it is what is defined and shown in Figure 2. “Seizures” needs to be defined in the results (Figure 1). Are “seizures” in the result section of Figure 1 “SWDs”? Higher temporal resolution recording is needed for Figure 1A, similar to Figure 2A.

3) The manuscript needs professional editing for content and format. It is difficult to read.

4) Specific ages of the rats were requested and ages are still not reported.

5) The “young” and “mature” categories for 3 or 5 months old rats have not been resolved. Please state the age rank (e.g., 3-4- vs. 5-6 month-old rats) to resolve this issue. Young and mature could have many meanings, particularly if the specific ages of the rats within each group are not provided. Do not use the term “approximately 14 weeks” or “above 20 weeks”, provide the exact rank of weeks to avoid confusion. Please include the specific ages of all rats and use age ranks to describe the groups.

6) High dose of ETX is still not defined. Please indicate the mg/kg used in the description of the results.

7) Define “depth” of the SWD in the text. The sentence is still not clear. “The incidence of spontaneous sleep spindles and SWDs with respect to the depth of the SWS (approximated by observing the density of delta waves, (Gervasoni et al., 2004)) was very different.”

8) Figure 1 is not clear since continuous recording were not performed. Please replot these graphs to clearly show that each data point is one week apart to clarify how the data was collected.

9) “Data are normalized. Can the authors include comparisons of data before normalization? Figure 2A, B” The reviewer was clearly refereeing to Figure 2C and D, where the data is normalized. Please address this.

10) “In E the units (1/s) are not clear for seizure occurrence rate.” The reviewer was clearly refereeing to the units of spindles. Please address this.

New specific comments:

11) The authors briefly mention causality when discussing the inverse relationship between spindle and seizure occurrences (figure 2E). This is troubling for two reasons: first, the authors have simply measured a correlation, but have not directly tested for causal relationships (or even defined causality). Second, to my understanding the authors did not correct for overall sleep changes in maturing rodents. Do non-epileptic rodents also show a decrease in sleep spindle occurrence during maturation, perhaps due to a gradual reduction in total sleep? If so, the correlation (and thus interpretation) of figure 2E may be explained by an un-accounted for factor (reduction in sleep quantity due to maturation). Is this relationship found within each rat as they mature or just represent rats with different levels of seizures?

12) Figure 2E, define ‘n’. Define the ‘n’ in each graph. ‘n’ is not the number of animals, which should be also reported, ‘n’ is the observations that are evaluated. I am assuming that each dot represents a week of recording for each rat (5 rats x 14 weeks of recording). There is no description of the ‘n’ for most of the figures.

13) There is a claim that SWDs did not occur during periods of activity, which is inferred via a negative correlation between SWD probability and hippocampal theta power (figure 3G-H). However, hippocampal theta is not restricted to periods of wakefulness. A more direct measurement of activity (video recordings) is necessary to support the above claim.

14) The linear regression applied in figure 3G-H is inappropriate on its own, as there is a strong oscillatory component in the residuals of data. The authors have already stated in their rebuttal that this is likely just random fluctuation, but this seems statistically unlikely as the data from both juvenile and mature rodents show similar oscillatory patterning.

15) Figure 4E does not seem to be representative of the population. Below (4F), the authors show a mean peak coherence between ispi and contra somatosensory cortex of roughly 0.7 early in development, although the example shown in 4E looks like it represents the upper tail of the distribution. A statistical test between in-focus and out-of-focus coherence should be applied (such as a 2-way repeated measures ANOVA).

16) The authors characterize well the transition probabilities into SWDs based on various sleep stages. However, these findings have been reported previously. For instance, Smyk et al. 2019 also found similar results using the WAGRij rat model of absence epilepsy. This doesn't negate the authors' efforts, and in fact it is even more convincing to see a replication of previous data; however the results presented in this study should be described in a broader context with appropriate citations so that novelty is de-emphasized.

17) The findings in figure 5 are difficult to interpret without seeing raw data. First, it is important to show the waveforms of the putative pyramidal and interneurons to illustrate the end-result of the methods used to discriminate these two neuron types. Second, the discussion of an altered inhibitory/excitatory balance simply based on firing rates of interneurons and pyramidal neurons is an estimation at best, and false information at worst. E/I balance is a complex subject that is influenced by many factors, and boiling this down to firing rates is a huge simplification. Finally, due to issues of spike-overlap during synchronous events (where action potentials become distorted due to synchronously firing neighboring neurons), the results presented in figure 5I are likely an underestimation. Without seeing the raw data with identified action potentials during a seizure, it is difficult to discern whether or not such data are actually representative of spiking, or simply reflect a limitation of spike-sorting methodology. In my opinion, panels F-G are the only convincing / novel results, as spike-phase relationship has not been characterized through development previously.

18) The results in figure 6 are intriguing, and provide a nice example of the progression of seizure generalization during development. It is unclear whether the data in panels D-I is representative of all animals used in this experiment, and should be made clearer.

19) Overall n's are quite low in this study. I understand the difficulty of the experiments, and I applaud the authors for taking on such a long experimental procedure. Nonetheless, because of the low sample sizes, statistical tests and reported p-values should be interpreted and presented more cautiously. Alternatively, the authors may want to replicate the results presented in figures 2 and 3 with another cohort of rodents.

20) The terms ‘juvenile’, ‘young’, ‘mature’, or ‘adult’ are still used for rats between 3 and 6 months. For example, the methods describe ‘young’ and ‘adult’ rats as ~3 and ~6 months, but does not provide age ranks (e.g. 2-4 or 4-7), or “>5 months”, without a clear limit. Please do not use “approximately” and give the exact rank. This lack of transparency regarding the age of the rats and lack of precision defining ages with the terms “juvenile” and ‘mature’ is a big concern that has not been resolved. To address this issue, please use the age group brackets to define the experimental groups (e.g., 3-4 months vs 5-6 months) instead of the poorly defined “juvenile” and “mature”.

21) Figure 1, despite the reviewer's comments, the n number and age of rats are still not clear. Are these 5 rats recorded from week 14 to week 28 (3 months) or these are two groups of rats (juvenile and mature = 5) graphing all data together? Please rewrite the text and legend to clarify this.

23) Figure 1E and 1F does not provide the n number or age. Figure shows “juvenile” and “mature” but does not describe the specific ages of the rats that were used to create these categories. It seems that only data at 3 and 6 months are plotted or are these numbers refereeing to the age group category? What are the n number for these of each experimental group?

24) Define the age groups with precision providing the age brackets, the term “~” has no meaning to understand the age. “We refer to ~14 weeks old animals as 'juvenile', while ages above 20 weeks were considered as 'mature' from here on.” Are these the same rats or different groups?

25) Line 246, “a substantial fall of spindle occurrence rate (Figure 2C) and duration (Figure 2D) was observed.” The graph does not show reduced spindle duration (don't understand the p value, p < 0.001). The occurrence rate does not show a “substantial” decrease since the variability increases significantly with age and the p value, p = 0.0562.

26) Figure 2, define what seizure is. It seems seizure = SWDs. If so, please use time in SWDs instead of “seizure” for all figure labels and text.

27) The ANOVA tests are not properly described. Factors, repeated measures, observations are not well defined.

28) Figure 2F, indicate what the y-axis is measuring. “% of control” does not mean anything. What is the control? Is the control baseline, before drug? Is the control a control rat? Is the control a vehicle injection? Be consistent. Text says “% of vehicle injection” graphs says “% of control”. Please include SWD and spindle into the y-axes to define the measure.

29) It is confusing that the text describes “seizures” but graphs plot sometimes “seizures” (Figure 2) and sometimes “SWD” (Figure 3). Please use “SWD” for all graphs and text, since SWD is what was defined in Figure 2A.

30) Figure 3A, please add the quantification of SWS to indicate the transition.

31) Line 277, Figure 3E, “seizures were very likely to occur when the animals entered SWS, but very few seizures were observed during SWS and during transitions from SWS.” Graph show seizures occur during “awake/REM”, not during ‘SWS’. Please define better the segments. “when the animal entered SWS” means that they are already in ‘SWS’. Please use what was defined “transition to SWS”.

32) Line 300, “~8Hz oscillations at 3 months (8.20 {plus minus} 2.10 Hz, Figure 4B), characteristic of spike-and wave-discharges.” Are these ‘SWD’ or just ‘8Hz’ oscillations? Please use the same term ‘SWD‘ through the result section. Using simultaneously “8Hz oscillations”, “seizures”, and “SWDs” does not help to understand the data and creates enormous confusion, which was already mentioned by previous reviewers.
